# Differential Effects of Endurance Exercise on Musculoskeletal and Hematopoietic Modulation in Old Mice

**DOI:** 10.14336/AD.2023.0713

**Published:** 2024-04-01

**Authors:** Zilin Wang, Hyun-Jaung Sim, Wenduo Liu, Jae Cheol Kim, Jeong-Chae Lee, Sung-Ho Kook, Sang Hyun Kim

**Affiliations:** ^1^Department of Sports Science, College of Natural Science, Jeonbuk National University, Jeonju 54896, Korea.; ^2^Department of Bioactive Material Sciences, Research Center of Bioactive Materials, Jeonbuk National University, Jeonju 54896, Korea.; ^3^Cluster for Craniofacial Development and Regeneration Research, Institute of Oral Biosciences and School of Dentistry, Jeonbuk National University, Jeonju 54896, Korea.

**Keywords:** Endurance training, Aging, HSC senescence, Musculoskeletal, Healthspan

## Abstract

One of the most important strategies for successful aging is exercise. However, the effect of exercise can differ among individuals, even with exercise of the same type and intensity. Therefore, this study aims to confirm whether endurance training (ETR) has the same health-promoting effects on the musculoskeletal and hematopoietic systems regardless of age. Ten weeks of ETR improved endurance exercise capacity, with increased skeletal muscle mitochondrial enzymes in both young and old mice. In addition, age-related deterioration of muscle fiber size and bone microstructure was improved. The expression levels of myostatin, muscle RING-finger protein-1, and muscle atrophy F-box in skeletal muscle and peroxisome proliferator-activated receptor-γ in the femur increased with age but decreased after ETR. ETR differentially modulated hematopoietic stem cells (HSCs) depending on age; ETR induced HSC quiescence in young mice but caused HSC senescence in old mice. ETR has differential effects on modulation of the musculoskeletal and hematopoietic systems in old mice. In other words, endurance exercise is a double-edged sword for successful aging, and great effort is required to establish exercise strategies for healthy aging.

## INTRODUCTION

Aging is a natural and unavoidable physiological process. Therefore, work toward strategies for successful aging of old individuals is increasing. The key to these efforts is exercise, which many studies have shown to improve health and healthspan [1]. Numerous studies have reported that exercise mimetics are highly effective for promoting health as well as preventing and treating metabolic diseases [2]. For the development of any medicine that mimics the effect of exercise, its mechanism and effect must be demonstrated through related studies.

The effect of exercise on the modulation of hematopoietic stem/progenitor cells (HSPCs) has been investigated. Exercise functions as an adjuvant therapy for hematopoietic stem cell mobilization and is currently used as a strategy for many cancer patients who require hematopoietic stem cell transplantation [3]. Exercise effectively modulates HSPCs by regulating hematopoietic output of inflammatory leukocytes in the bone marrow (BM) microenvironment [4]. Hematopoiesis and HSPCs are differentially modulated depending on the exercise protocol [5].

A training program aimed at improving health and healthspan should be structured according to FITT (frequency, intensity, time, type) principles as well as basic principles such as exercise specificity and individual variations [6]. Physical activity following an appropriate training program affects life expectancy. In particular, endurance training (ETR), which is defined according to the basic principles of exercise, is an essential factor in the health, quality of life, and life expectancy of older adults [7]. Many studies have encouraged people to continuously engage in endurance exercise from a young age or to actively participate in an ETR program [8]. However, oxidative stresses such as reactive oxygen species (ROS) increase after a single bout of exercise, and thus exercise is also a stress applied to the body [9]. In other words, excessive exercise or overtraining poses a health risk [10,11]. The threshold for excessive exercise varies among individuals. Even for individuals of the same sex and weight, the relative intensity of exercise varies depending on body composition and other factors.

This study aims to confirm whether ETR using a treadmill has the same health-promoting effect on modulation of the musculoskeletal and hematopoietic systems regardless of age.

## MATERIALS AND METHODS

### Experimental animals

Young (4-week-old) and old (15-month-old) C57BL/6J male mice were housed (n = 4-5 per cage) under controlled temperature (18-22°C) and humidity (40-60%) with a 12-h light/dark cycle; food and water were provided ad libitum. The lower leg muscles (gastrocnemius, GAS; soleus, SOL; tibialis anterior, TA; extensor digitorum longus, EDL) of the left side were isolated at 18 h after the last exercise bout. The muscles were rapidly frozen and stored at -80°C until western blot analysis. Bones (femur and tibia) and lower leg muscles of the right side were extracted and fixed for histological and immunohistochemical staining. The weight of the removed tissues was measured using an electronic balance. The present study received approval from the Institutional Animal Care and Use Committee of Jeonbuk National University (IACUC approval no. CBNU-2022-0067).

### Endurance training protocol

All mice were trained for ten weeks after acclimatization to treadmill running for 20 min at 6-10 m/min, repeated three times. Endurance capacity was measured before ETR, confirming that old mice had 15% to 20% lower exercise capacity than young mice. Based on this result, the exercise volume for old mice was set to approximately 80% of that for young mice by adjusting the running speed. On a treadmill with a 0% incline, the running speed for young mice was set to 60-70% of the maximum running speed based on a related study [12]. ETR consisted of warm-up, main exercise, and cool down periods, and was conducted four times per week for a total of 60 min each time. The warm-up for young mice was performed at a speed of 10 m/min for 10 min, followed by running at a speed of 15 m/min for 45 min. Cool down was performed for 5 min at a speed of 10 m/min. For old mice, after a warmup at 8 m/min, treadmill running was continued at a running speed of 12 m/min, followed by cooling down at 8 m/min. If the mice remained on an electric shock grid that did not conduct electricity while running on the treadmill, the tail and hind limbs were stimulated using a sponge. After ten weeks (4 bouts per week) of ETR, an endurance exercise test was conducted to verify the effect of training.

In the progressive exercise test for endurance exercise capacity, subjects ran at a speed of 10 m/min for the first 5 min on a treadmill set at a 15° incline, after which the speed increased by 2 m/min every minute. When each mouse could no longer run and remained on an electric shock grid where no current was applied, mice were stimulated to continue running by stimulating their tails and hind limbs with a sponge. The test was stopped if the mouse remained on the grid for more than 3 seconds despite the sponge stimulation. The total running time and vertical distance were measured as described previously [13]. Work was calculated using a formula reported previously [14].

### Measurement of plasma osteocalcin and indicators of oxidative stress

The plasma levels of osteocalcin (OC) and thiobarbituric acid-reactive substances (TBARs) were analyzed from the experimental mice by colorimetric assays. Briefly, whole blood samples were collected from the heart of mice 18 h after the last exercise bout. The samples were centrifuged in heparinized tubes to separate the blood plasma samples. The plasma samples were stored at -80°C prior to further analysis. Measurement of plasma OC levels was conducted using an enzyme-linked immunosorbent assay (ELISA) kit (Elabscience Biotechnology Co., Ltd., Wuhan, China) as described previously [15]. The plasma level of TBARs was fluorometrically determined using the Quantichrom TBARS Assay Kit (Bioassay Systems, Hayward, CA, USA) as previously outlined [16].

### Western blot analysis

The GAS muscle, obtained from the skeletal muscles 18 h after the last exercise bout, was homogenized in a buffer at 4°C. The buffer consisted of the following components: 50 mM Tris·HCl (pH 7.4), 1% NP-40, 0.25% sodium deoxycholate, 150 mM NaCl, 1 mM ethylenediaminetetraacetic acid (EDTA, pH 7.4), 1 mM Pefabloc (Roche, Basel, Switzerland), 1 mM NaF, 1 μg/ml of aprotinin, leupeptin, and pepstatin, 0.1 mM bpV (phen), and 2 mg/ml β-glycerophosphate. The protein concentrations were determined using the Lowry method [17]. The homogenate was solubilized in Laemmli sample buffer and subjected to sodium dodecyl sulfate-polyacrylamide gel electrophoresis before being transferred to nitrocellulose membranes. Following a 1-h block at room temperature, the membranes were incubated overnight at 4°C with the following antibodies: β-actin (Ma1-140, Invitrogen, Carlsbad, CA, USA), growth differentiation factor-8 (GDF8)/myostatin (sc-393335, Santa Cruz Biotechnology, Santa Cruz, CA, USA), muscle-specific RING finger protein (MuRF-1, sc-398608, Santa Cruz Biotechnology), and muscle atrophy F-box (Mafbx, sc-166806, Santa Cruz Biotechnology). Subsequently, the blots were incubated with appropriate secondary antibodies. The detection of antibody-bound proteins was performed using ECL Western Blotting Detection Reagent (GE Healthcare, Chalfont St Giles, UK), and quantification was carried out with the ChemiDoc XRS+ system (BIO-RAD, Hercules, CA, USA).

### Histological analyses

Bone (femur and tibia) and muscle samples (TA and GAS) were prepared for histological analysis following previously established protocols [15]. The tissues underwent decalcification utilizing 10% EDTA at room temperature. After dehydration with ethanol, each tissue was clarified with xylene, infiltrated, and embedded in paraffin. The embedded tissues were sectioned and stained with hematoxylin and eosin (H&E) on glass slides. The Motic Easy Scan One slide scanner (Meyer Instruments, Inc., Houston, TX, USA) was used to observe the sizes of muscle fibers, intermuscular adipose tissue (IMAT), and BM adipose tissue (BMAT), which were further quantified using ImageJ software (ver 1.51, NIH, Bethesda, MD, USA).

Immunohistochemistry (IHC) was conducted following the methods described previously [15]. Tissue sections were stained with each of primary antibodies; peroxisome proliferator-activated receptor γ (PPAR-γ, sc-7273), receptor activator of nuclear factor kappa-B (RANK, sc-374360), RANK ligand (RANKL, sc-377079), and osteoprotegerin (OPG, sc-390518) that were purchased from Santa Cruz Biotechnology. The VECTASTAIN Universal Elite ABC Kit (Vector Laboratories, Burlingame, CA, USA) and DAB Substrate kit (Vector Laboratories) were utilized for the detection of proteins through the IHC staining. Digital images of the stained tissues were captured using the Motic Easy Scan One slide scanner (Meyer Instruments, Inc.). These images were subjected to quantitative analysis for the positive area, integrated optical density, and mean optical density using Image Pro Plus 6 (Media Cybernetics Inc., Rockville, MD, USA).

### Micro-computed tomography analysis

Following ten weeks of ETR, the mice were euthanized, and the right-side leg was preserved in a solution of 10% formalin. To evaluate the bone microstructure and quality, the samples were analyzed by micro-computed tomography (micro-CT) using the SkyScan 1076 system (Bruker, Kontich, Belgium) at the Center for University-Wide Research Facilities of Jeonbuk National University as described previously [15]. The images were constructed in two dimensions using NRecon version 1.3 (SkyScan) followed by three-dimensional (3D) reconstruction utilizing the CTAn and CTVox software (SkyScan). For the analysis of the 3D images, regions of interest (ROIs) were defined in the CTAn software, comprising 150 slides at a distance of 0.5 mm from the growth plate. Based on the reconstructed 3D images, values of bone mineral density (BMD, mg/cc), bone/tissue volume (BV/TV, %), bone surface area/bone volume (BS/BV, 1/mm), trabecular thickness (Tb.Th, μm), trabecular number (Tb.N, 1/mm), and trabecular separation (Tb.Sp, μm) were determined.

### Flow cytometry

The numbers and phenotypes of BM cells in experimental mice were determined using multi-color flow cytometer (BD Aria, Franklin Lakes, NJ, USA) installed in the Center for University-Wide Research Facilities of Jeonbuk National University. The populations of hematopoietic lineage cells in the BM were phenotypically gated and analyzed using FlowJo software (FLOWJO; Ashland, OR, USA). Lin^-^Sca-1^+^c-Kit^+^ (LSK) cells and HSCs were defined using the following antibodies (purchased from BD Biosciences, unless otherwise specified): lineage markers phycoerythrin (PE)-Cy7-conjugated anti-CD3 (#552774), anti-CD4, anti-CD8, anti-CD45R (#552772), anti-CD11b (#552850), anti-Gr-1 (#552958), and anti-TER-119 (#557853); PE- (#553108) or fluorescein isothiocyanate (FITC)-conjugated anti-stromal cell-derived factor 1 (Sca-1, #557405); allophycocyanin (APC)-conjugated anti-c-Kit (#553356); PerCP/Cy5.5-conjugated anti-CD150 (#46-1502, eBioscience, Waltham, MA, USA); and APC-Cy7-conjugated anti-CD48 (#561826). BM-derived HPCs (e.g., granulocyte-monocyte progenitors, GMP; common myeloid progenitors, CMP; megakaryocyte-erythroid progenitors, MEP; common lymphoid progenitors, CLP) were characterized using PE-conjugated anti-FcR and PerCP/Cy5.5-conjugated anti-CD34 on the basis of Lin^-^Sca-1^-^c-Kit^+^ markers, as well as PE-conjugated anti-IL-7R. Proportions of circulating monocytes (CD11b^+^) and B-cells (B220^+^) in the peripheral blood (PB) were measured using the FITC- and PE-conjugated antibodies, respectively. The mitochondrial superoxide anion level and senescence-associated beta-galactosidase (SA-β-gal) activity was measured with MitoSox Red (#M36008, Invitrogen) and 5-dodecanoylaminofluorescein di-β-D-galactopyranoside (#I2904, C_12_FDG; Molecular Probes, Eugene, OR, USA), respectively.

### Transplantation experiment

To evaluate donor cell-derived repopulating capacity, an equal number of LSK cells (5 × 10^3^) from sedentary (SED) or ETR mice (CD45.2) were co-transplanted with equal numbers of LSK cells from competitor mice (CD45.1) and accessory PB cells (2 × 10^6^) from non-conditioned recipient mice (CD45.1/2) via tail vein injection into conditioned recipient mice (CD45.1/2), which had been lethally irradiated (9 Gy) 8-24 h prior to transplantation. The transplanted donor cell-mediated repopulating capacity was measured in the PB of the recipient mice at 4 months post-transplant. To determine the resistance of donor cells to genotoxic stress, the recipient mice were sub-lethally irradiated (5 Gy). The repopulating and engraftment capacities of the donor cells were assessed based on the PB and BM of the recipient mice, respectively, at 2 months after sub-lethal irradiation.

### Real-time reverse transcription polymerase chain reaction (RT-PCR)

Total RNA was extracted with TRIzol reagent (Invitrogen) according to the manufacturer’s instructions. RNA samples (1 µg per reaction) from each group were used for preparation of cDNA for RT-PCR using the AmpiGene^TM^ cDNA Synthesis Kit (Enzo Life Sciences, Farmingdale, NY, USA). Power SYBER^®^ Green PCR Master Mix (Life Technologies, Carlsbad, CA, USA) was used to detect the accumulation of PCR products during cycling with the ABI StepOnePLUS sequence detection system (Applied Biosystems, Foster City, CA, USA) with the following program: pre-denaturation at 95°C for 2 min followed by 40 cycles of denaturation at 95°C for 5 s, annealing at 60°C for 30 s, and extension at 65°C for 30 s. Primers specific to *p16* (forward-gtcgcaggttcttggtcact and reverse-tctgcaccgtagttgagcag) and *p21* (forward-tgtccgtcagaacccatc and reverse-aaagtcgaagttccatcgcc) were designed using Primer Express 3.0 (Applied Biosystems). Glyceraldehyde 3-phosphate dehydro-genase (*Gapdh*, forward-gacggccgcatcttcttgt and reverse-cacaccgaccttcaccatttt) was used as an endogenous reference for quantification.

### Statistical analyses

All data are expressed as the mean ± standard deviation and were analyzed using GraphPad Software (Prism 9, Boston, MA, USA). Differences between two groups were analyzed by unpaired Student’s *t*-test (n ≥ 6) or by a non-parametric test (Wilcoxon *t*-test, n < 6). Two-way ANOVA followed by the Tukey post hoc test was used for multiple comparisons among more than two groups. The Kolmogorov-Smirnov test was used to test the normality of data sets. A value of *p* < .05 was considered statistically significant.

## RESTULTS

### Endurance training improves aging-related musculoskeletal disorders

**Figure 1.**
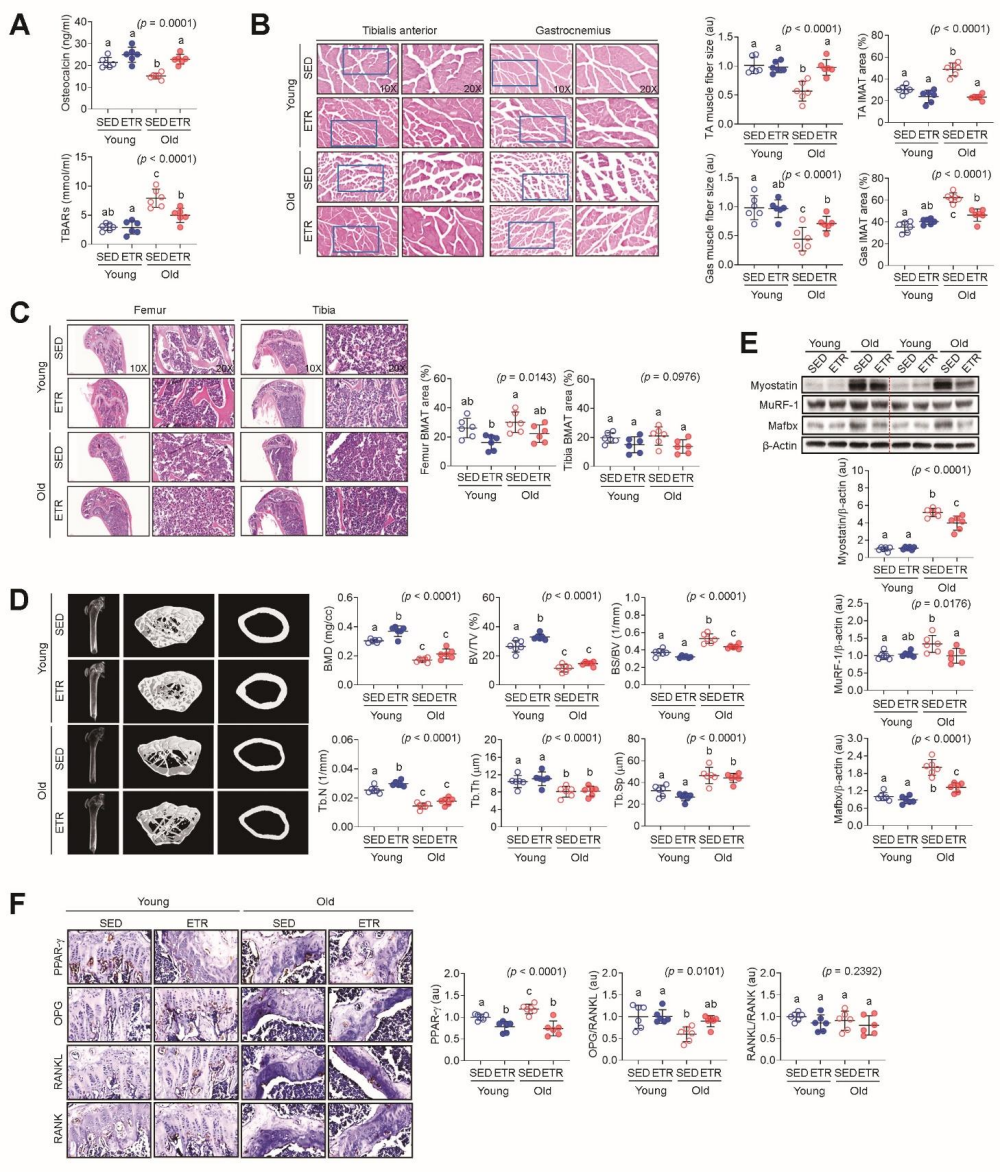
**ETR for ten weeks improves age-associated musculoskeletal disorders**. **(A)** Levels of OC and TBARs in the plasma were compared between the groups (n = 6). **(B)** Muscle fiber size and IMAT area in TA and GAS were analyzed by H&E staining, and their relative values (au or %) were compared between the groups (n = 6). **(C)** H&E staining images showing BMAT area (%) in femur and tibia in the experimental groups along with the statistical comparison between the indicated groups (n = 6). **(D)** Bone microstructures in long bones of mice groups were analyzed by micro-CT, and values of bone-specific parameters were compared between the groups (n = 6). **(E)** Protein levels of myostatin, MuRF-1, and Mafbx in gastrocnemius muscles were analyzed by western blotting, and the immunoreactive band intensities were compared between the groups (n = 6). **(F)** Expression levels of adipogenic and bone remodeling-related molecules in femoral sections were analyzed by IHC and compared between the groups (n = 6). Representative images expressing an average morphology or intensity among the samples in the same group are shown in panels B-F, respectively. Different letters (a-c) indicate significant differences among the groups in the indicated *p* values according to two-way ANOVA followed by Tukey post hoc test.

Changes in the musculoskeletal system, such as muscle atrophy and decreased BMD, are associated with aging [18]. Several metabolism-related blood variables were affected by aging. Among them, TBARs, which are formed as a byproduct of lipid peroxidation, and OC, an indicator of bone metabolism, are closely associated with aging [19]. In this study, the OC level was significantly lower in old SED than young SED mice (Fig. 1A), but the TBAR level was significantly higher in the old SED group (Fig. 1A). These negative changes associated with aging improved the levels of young SED mice with ETR. For OC, both old and young mice showed increases with ETR.

Skeletal muscles and bones exhibit both spatial and metabolic interconnections facilitated by signaling molecules, including myokines (such as myostatin, IL-6, irisin, etc.) and osteokines (such as osteocalcin, sclerostin, FGF23, etc.), respectively [20]. These tissues show similar changes throughout life. In general, the quantity and quality of both begin to decline after middle age [21]. In this study, the muscle fiber sizes (Fig. 1B) of the TA and GAS were significantly smaller in the SED old group than the SED young group. More IMAT (Fig. 1B) was also observed in the SED old group than the SED young group. Contrary to expectations, there was a slight increase in IMAT of the GAS muscle following exercise, but no significant difference was observed (Fig. 1B). These negative changes in the SED old group demonstrated improvements, such as increases in muscle mass and fiber size and a decrease in IMAT with ETR, which were not observed in the SED young group.

Similar to skeletal muscle, bone showed negative changes in older mice. In terms of BMAT, SED old was not greater than SED young, but ETR significantly decreased femoral BMAT in both young and old mice (Fig. 1C). BMD and bone microstructure measured using micro-CT showed negative changes in all measurements of SED old mice compared to SED young mice (Fig. 1D). These negative changes were significantly improved by ETR in the old group in terms of BMD, Tb.N, BV/TV, and BS/BV values, and in the young group in terms of BMD, Tb.N, BS/BV, and Tb.Sp values.

We performed immunoblotting to determine whether ETR regulates the modulators related to musculoskeletal function and morphology. The expression levels of proteins related to skeletal muscle atrophy including myostatin, muscle MuRF-1, and Mafbx were significantly elevated in SED old mice compared to SED young mice. In the GAS muscle, an effect of ETR was observed in old, but not young, mice (Fig. 1E). The results from IHC in combination with hematoxylin staining revealed that PPAR-γ was significantly elevated in SED old mice compared to SED young mice, but decreased with ETR regardless of age (Fig. 1F). By contrast, OPG/RANKL, which determines bone mass and skeletal integrity[22], showed a significant decrease in SED old mice compared to SED young mice, but was significantly increased by ETR. RANKL/RANK signaling, which regulates osteoclastogenesis, showed no significant difference in any comparison (Fig. 1F).

**Figure 2.**
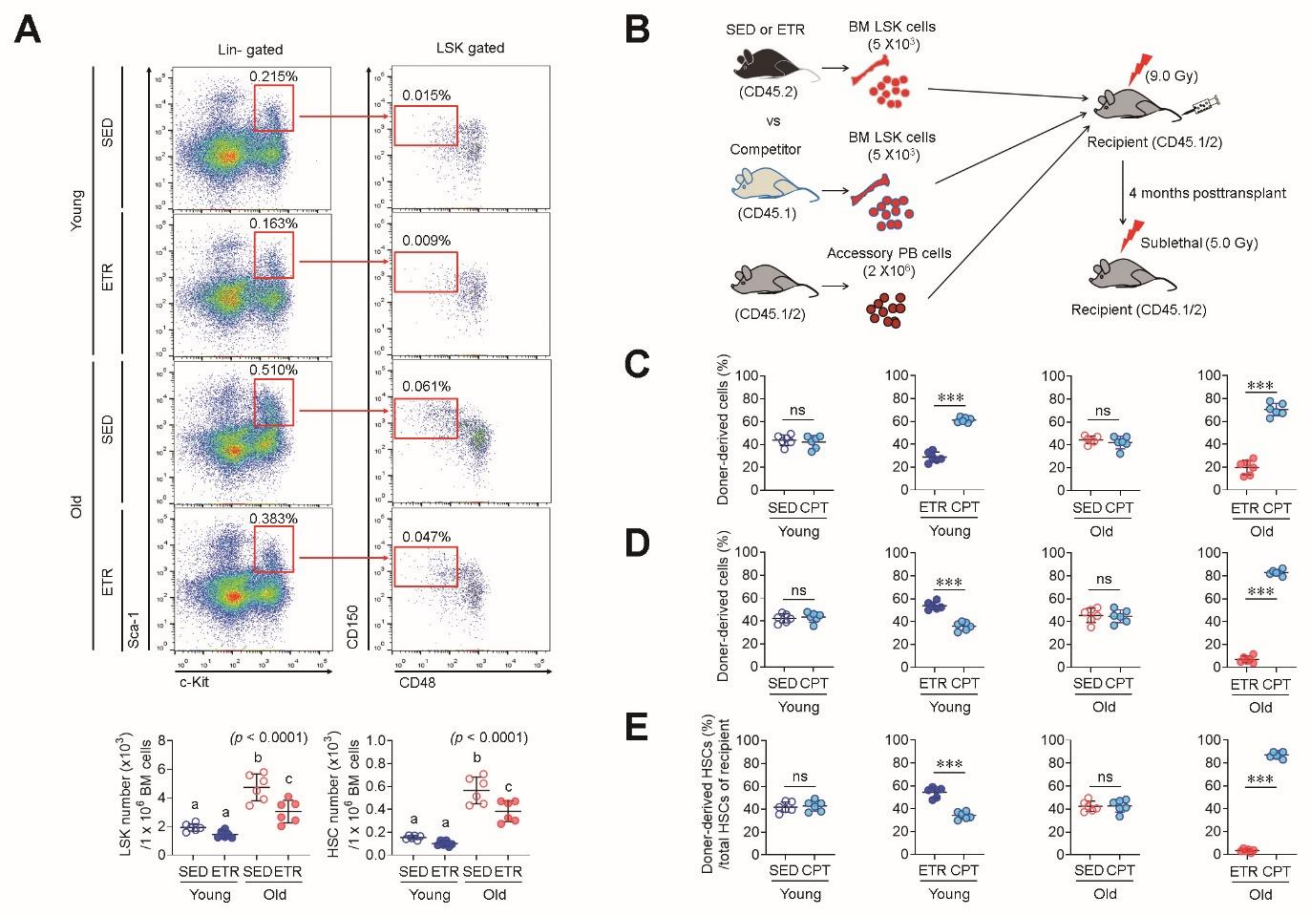
**ETR differentially modulates HSPC function depending on age**. **(A)** LSK cells and HSCs in the BM were phenotypically defined as Lineage^-^Sca-1^+^c-Kit^+^ and CD150^+^CD48^-^LSK cells, and their numbers were measured using multi-color flow cytometry (n = 6). Different letters (a-c) indicate significant differences among the groups in the indicated *p* values according to two-way ANOVA followed by Tukey post hoc test. **(B)** Schematic diagram of transplantation experiments conducted for analysis of competitive repopulating potential and engraftment capacity. BM LSK cells from SED or ETR mice (CD45.2) were co-transplanted with equal numbers (5 × 10^3^) of BM LSK cells from competitor mice (CD45.1) and accessory cells (2 × 10^6^) from PB of non-conditioned recipient mice (CD45.1/2) into the conditioned recipient mice (CD45.1/2, 900 rads) that had been lethally irradiated. The age of CD45.1 and CD45.2 mice used for the transplantation was similar. **(C)** For analysis of donor cell-derived repopulating capacity, the CD45.1/CD45.2 ratio in the PB of the recipient mice was measured at 4 months post-transplantation (n = 6). **(D)** Transplanted donor cell-derived reconstituting capacity was analyzed in conditioned recipients exposed to S-TBI (500 rads) at 4 months post-transplantation (n = 6). **(E)** For analysis of engraftment potential, the percentage of HSCs was measured in the BM of S-TBI-exposed recipient mice (n = 6). In panels C-E, *p* values indicate significant differences in ^*^p < 0.05, ^**^p < 0.01, or ^***^p < 0.001 between the groups by unpaired Student's *t*-test. ns, not significant. CPT, Competitor.

### ETR differentially modulates HSPC function depending on age

To determine the effect of ETR on the modulation of BM-conserved HSPCs in relation to age, we analyzed the number of these cells using multi-color flow cytometry. In accordance with a previous study indicating that aged mice had a greater number of HSPCs than young mice [23], SED old mice exhibited significantly higher numbers of LSK cells and HSCs than SED young mice (Fig. 2A). BM cells from ETR young mice showed a significant decrease in the number of LSK cells and HSCs compared to SED young mice (Fig. 2A). Similarly, the numbers of LSK cells and HSCs were significantly reduced in the BM of ETR old mice compared to SED old mice (Fig. 2A).

To assess HSPC function, we conducted competitive transplantation through co-transplanting equal numbers of LSK cells (5 × 10^3^) from SED or ETR mice (CD45.2) and competitor mice (CD45.1) into conditioned recipient mice (CD45.1/2) that were also transplanted with accessary PB cells (2 × 10^6^) from non-conditioned recipient mice to improve survival of conditioned recipients (Fig. 2B). The transplantation results showed that LSK cells from SED young mice could compete with those from competitor young mice at 4 months post-transplantation (competitor, 42.1%; SED young, 43.8%, Fig. 4C). However, LSK cells from ETR young mice were outcompeted by those from competitor young mice in the PB of the recipient mice (competitor, 61.3%; ETR young, 28.9%, Fig. 2C). Similar to young mice, LSK cells from SED old mice could compete with those from competitor old mice (competitor, 42.1%; SED old, 44.4%, Fig. 2C). By contrast, LSK cells from ETR old mice had poor repopulating capacity in the recipient mice (competitor, 70.4%; ETR old, 19.7%) compared to cells from competitor mice and exhibited more deficient repopulating capacity than LSK cells from ETR young mice.

The lower repopulating capacity of ETR-derived LSK cells in recipient mice after transplantation may be associated with cell quiescence or senescence caused by ETR. Previous research has demonstrated that exercise enhances HSPC quiescence. As quiescent HSCs are more resistant to genotoxic stress [24], we exposed the transplanted recipient mice to sub-lethal total body irradiation (S-TBI) to determine whether the poor repopulating capacity of ETR-derived LSK cells in the recipient mice was due to ETR-mediated cell quiescence (Fig. 2B). SED-derived young LSK cells exhibited comparable reconstituting capacity with competitor-derived young LSK cells in S-TBI-exposed recipient mice (competitor, 42.9%; SED young, 41.9%, Fig. 2D). In contrast to the transplanted recipient mice, ETR-derived young LSK cells of S-TBI-exposed recipient mice were able to outcompete competitor-derived young LSK cells (competitor, 34.1%; ETR young, 54.4%, Fig. 2D). Notably, ETR-derived old LSK cells showed a lower repopulating capacity in S-TBI-exposed recipient mice than in the initial transplant recipient mice (competitor, 87.1%; ETR old, 3.4%, Fig. 2D), in contrast to the result in ETR-derived young LSK cells. The reconstituting capacity of ETR-derived young and old LSK cells was reflected in the number of HSCs engrafted in the BM of S-TBI-exposed recipient mice, in that significantly controversial impacts to the corresponding competitors were found in HSCs derived from ETR, but not SED, young and old mice. These results suggest differing effects of ETR on the modulation of HSPC function depending on age.

### Endurance training leads to HSC senescence in old mice

**Figure 3.**
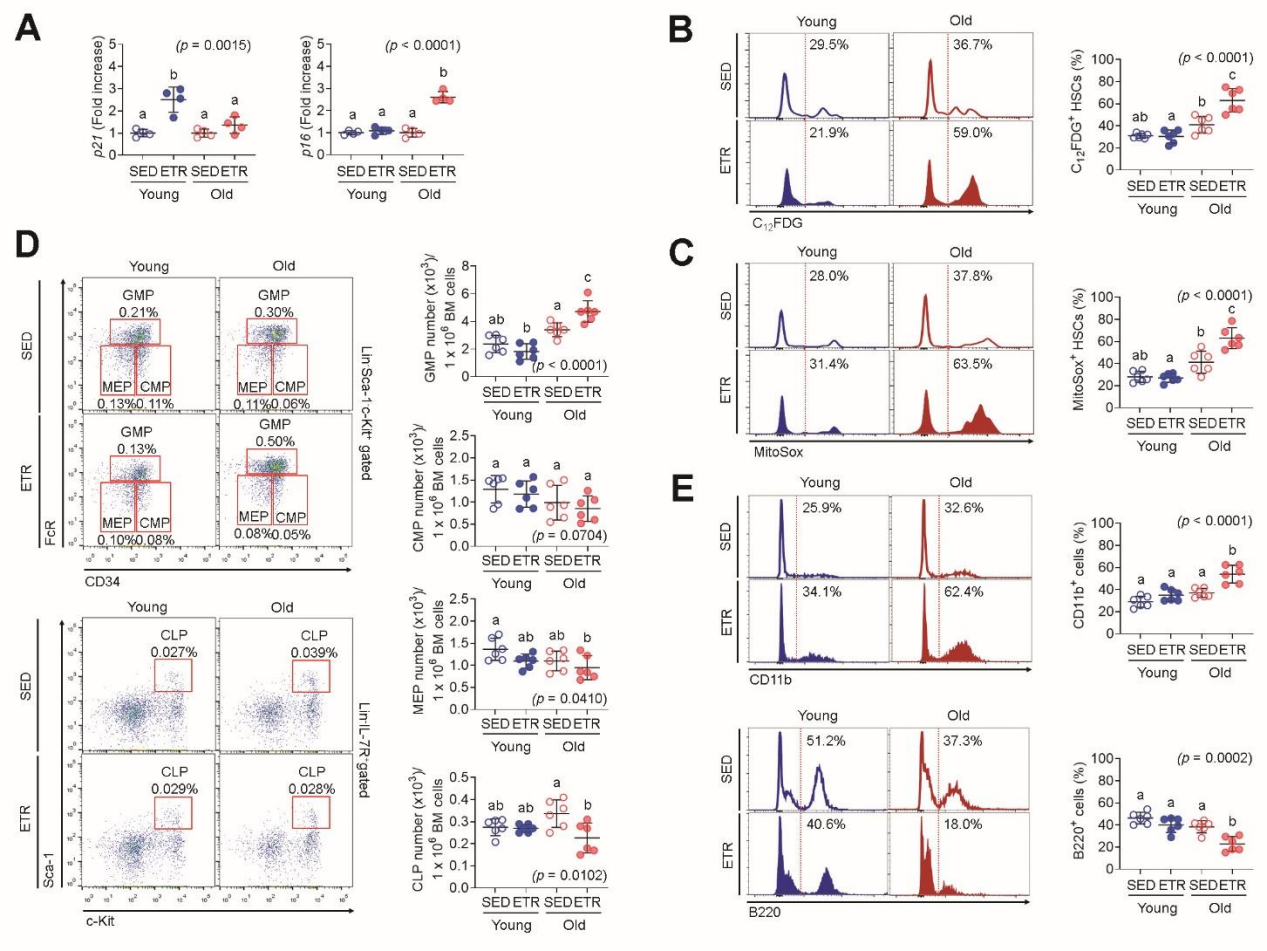
**ETR renders HSCs senescent in old mice**. **(A)** Levels of *p21* and *p16* in LSK cells that were sorted from the BM of SED and ETR mice using flow cytometry were measured by RT-PCR (n = 4). Levels of (B) SA-β-gal activity and (C) mitochondrial superoxide anions in BM HSCs were determined by flow cytometry using C_12_FDG and MitoSox Red, respectively (n = 6). **(D)** The frequencies of GMPs, CMPs, MEPs, and CLPs were measured in BM of the groups by flow cytometry (n = 6). **(E)** The percentages of circulating lymphoid (B220^+^) and myeloid (CD11b^+^) lineages were also measured in the PB of the groups by flow cytometry (n = 6). Different letters (a-c) indicate significant differences among the groups in the indicated *p* values according to two-way ANOVA followed by Tukey post hoc test.

To determine the mechanism through which ETR exerts a differential impact on the modulation of HSC function depending on age, we first measured mRNA levels of cyclin-dependent kinase inhibitors such as *p21* and *p16*, which are important genes for cell quiescence and senescence, respectively [25-27]. ETR young mice had significantly increased mRNA levels of *p21* in LSK cells, while ETR old mice exhibited no change in *p21* mRNA in LSK cells relative to the corresponding SED mice (Fig. 3A). This result reflects a lower reconstituting capacity in the conditioned recipient mice transplanted with ETR-derived young LSK cells and outcompeting capacity in the conditioned recipient mice after exposure to S-TBI. In contrast to *p21*, mRNA levels of *p16* increased significantly in ETR old mice, but not in ETR young mice, compared to the corresponding SED mice (Fig. 3A). As reduced HSC function is associated with cell senescence [28], we further assessed the extent of senescence in HSCs using C_12_FDG, a β-galactosidase substrate. As shown in Fig. 3B, the activity of SA-β-gal was greatly enhanced in SED or ETR old mice compared with their corresponding young mice, respectively (Fig. 3B). Comparing the SA-β-gal activity between SED and ETR old mice, the mice with ETR exhibited significantly higher activity than that with SED (Fig. 3B). As a stem cell senescence is accelerated under the influence of ROS [29,30], we measured the level of mitochondrial ROS in HSCs using MitoSOX. Similar to the SA-β-gal activity, old mice showed greater level of MitoSox-positive HSCs (%) than young mice regardless of SED or ETR, in which the ETR old mice exhibited the highest accumulation of mitochondrial ROS in HSCs among the mice groups (Fig. 3C).

The differentiation potential of senescent HSCs becomes more biased toward myeloid lineages and away from lymphoid lineages [31]. Compared with SED young mice, ETR young mice did not show any changes in the BM numbers of GMPs, CMPs, MEPs, and CLPs (Fig. 3D). However, the BM of ETR old mice contained significantly higher GMP and fewer CLP cells without changes in the populations of CMP and MEP cells compared with SED old mice (Fig. 3D). Similarly, the PB of ETR old mice showed a bias toward myeloid-lineage (CD11b^+^) cells rather than lymphoid-lineage (B220^+^) cells relative to the PB of SED old mice (Fig. 3E). Meanwhile, ETR young mice exhibited no alteration in these lineages of cells in the PB relative to SED young mice (Fig. 3E). Taken together, these findings suggest that ETR differentially affects HSCs, rendering them quiescent in young mice and senescent in old mice.

## DISCUSSION

Skeletal muscles and bones, which play vital roles in physical activity, are key tissues involved in improving healthspan and reducing the risk of premature mortality. In particular, skeletal muscle contraction induces a mechanical stimulus to the bone as well as the expression or activity of various proteins to maintain the health of skeletal muscle cells and BM [32]. Alternatively, physical training involving skeletal muscle contraction induces a highly complex biological response that induces interactions among different genes within various cells, organs, and tissues, with diverse effects including health promotion and healthspan extension. Therefore, this study aimed to assess the benefit of ETR in terms of health promotion and longevity regardless of age.

Aging-related musculoskeletal disorders are common problems affecting the elderly and are associated with decreased muscle strength, increased bone fragility, and fat redistribution [33,34]. In this study, reduction in muscle fiber size, increase in IMAT, decrease in BMD, and negative changes in bone microstructures were confirmed (Fig. 1A-D). In addition to these morphological changes, the expression of ubiquitin proteasome system genes, MuRF-1 and MAFbx, which are one of the key mechanisms related to muscle atrophy in skeletal muscle [35], and myostatin, which negatively regulates muscle mass as a member of transforming growth factor beta [36], were increased (Fig. 1E). In bone tissue, PPAR-γ expression related to adipose tissue production [37,38] increased and OPG/RANKL ratio decreased (Fig. 1F). An increase in OPG/RANKL ratio means inhibition of osteoclast production [39,40]. It was confirmed that these negative changes in old mice were improved by 10 weeks of ETR. Exercise exerts its effects through substrate utilization and enzyme activation through very complex mechanisms involving various metabolic and molecular changes [41]. Therefore, it can be seen that the effect of ETR on improving the negative changes caused by aging confirmed in this study is due to inhibition of skeletal muscle atrophy and inhibition of adipogenesis and osteoclast production in bone tissue.

Exercise has numerous undeniable benefits for health and healthspan. However, some studies indicate that exercise is harmful, rather than helpful, for health promotion [42-44]. In other words, the effect of exercise can vary depending on the exercise protocol. As demonstrated in this study, ETR can improve musculoskeletal disorders associated with aging, although aging of the hematopoietic system is simultaneously accelerated. This study verified the effects of 10 weeks of endurance training via treadmill running, but did not test the effects of exercise intensity, type, duration, or other factors. In addition, in this study, aging of the hematopoietic system of young mice did not progress after ETR, and thus the timing of the first period of exercise in life must be considered important. Therefore, for successful aging, maintaining regular and continuous exercise from adolescence is essential. In the future, effects associated with the FITT plan (frequency, intensity, type, time) for physical activity by the elderly mice must be verified.

Exercise renders HSCs quiescent in young mice [4]. Quiescent HSCs possess long-term repopulating potential and engraftment capacity after transplantation, and have the capacity for high resistance to stresses such as transplantation and genotoxic agents [45,46]. Similarly, ETR young mouse-derived HSCs exhibited quiescence-associated phenotypes, as evidenced by higher levels of p21 mRNA (Fig. 3A) and had superior transplant characteristics (Fig. 2C-E). In contrast to the positive effect of exercise on modulating HSCs in young mice, HSCs of old mice responded negatively to the same exercise protocol. ETR caused HSCs to become senescent, as evidenced by higher levels of senescence-related factors such as SA-β-gal activity, mitochondrial ROS, and p16 mRNA (Fig. 3A-C), as well as myeloid-biased differentiation (Fig. 3D-E) and defects in competitive reconstituting potential and radioprotection (Fig. 2C-E). Our current findings illustrate differential effects of endurance exercise on the modulation of HSCs depending on age. In other words, exercise strategies must be established with consideration of age for effective maintenance of the HSC-based hematopoietic system, in contrast to the musculoskeletal system.

Exercise is widely acknowledged to offer numerous undeniable benefits for both health and lifespan [47]. However, the exercise protocol employed in this study led to the induction of senescence in hematopoietic cells of aged mice, with the precise underlying causes yet to be determined. It is important to recognize that exercise can be a double-edged sword [48-50], potentially posing risks to health rather than promoting it [38-40]. A key argument in support of this notion is that excessive intensity or volume of exercise may not contribute to enhanced health outcomes. When considering aging mice with relatively lower fitness levels, it becomes crucial to engage in exercise at lower intensities or volumes. Therefore, it is imperative to recommend a safe level of exercise program, taking into account factors such as age, although further research in this area is required.

In summary, this study verified the effects of 10 weeks of endurance training via treadmill running. This study highlights that differently to old mice, the hematopoietic system in young mice was not affected after ETR, and thus the timing of the first period of exercise in life must be considered importantly. This indicates that for successful aging, maintaining regular and continuous exercise from adolescence is to be essential. Based on the current findings showing the impacts of exercise resembling a double-edged sword in relation to age, it might be that the optimal intensity and volume of exercise are recommended for elderly people. Further experiments to elucidate the age-associated physical effects of exercise regarding to the FITT plan (frequency, intensity, type, and time) will be necessary.
